# A care ethical perspective on family caregiver burden and support

**DOI:** 10.1177/09697330251324294

**Published:** 2025-03-04

**Authors:** Maaike Haan, Jelle van Gurp, Marianne Boenink, Gert Olthuis

**Affiliations:** 6034 Radboud University Medical Center; 6034 Radboud University Medical Center; 6034 Radboud University Medical Center; 6034 Radboud University Medical Center

**Keywords:** caregiver burden, caregiver support, ethics of care/care ethics, family care, informal care, palliative care

## Abstract

Family care—when partners, relatives, or other proxies care for each other in case of illness, disability, or frailty—is increasingly considered an important pillar for the sustainability of care systems. For many people, taking on a caring role is self-evident. Especially in a palliative care context, however, family care can be challenging. Witnessing caregivers’ challenges may prompt compassionate nurses to undertake actions to reduce burden by adjusting tasks or activities. Using a care ethical approach, this theoretical paper aims to provide nurses with an alternative perspective on caregiver burden and support. Drawing on the concepts of relationality and contextuality, we explain that family care often is not a well-demarcated or actively chosen task. Instead, it is a practice of responding to an all-encompassing “call” to care flowing from a relationship, within a social and cultural context where norms, motivations, and expectations shape people’s (sometimes limitless) care. We consider relational interdependence at the root of persisting in care provision. The question is then whether self-sacrifice is a problem that nurses should immediately solve. In ideal circumstances, self-sacrifice is the result of a conscious balancing act between values, but family care in the context of serious illness barely provides room for reflection. Yet, instant attempts to alleviate burden may overlook family caregivers’ values and the inherent moral ambiguities and/or ambivalent feelings within family care. Family care is complex and highly personal, as is finding an adequate balance in fulfilling one’s sometimes conflicting values, motivations, and social expectations. Therefore, we suggest that caregiver experiences should always be interpreted in an explorative dialogue, focused on what caring means to a particular family caregiver. Nurses do not have to liberate family caregivers *from* the situation but should support them *in* whatever overwhelms or drives them in standing-by their loved ones until the end.

## Introduction

Family care—when partners, relatives, or other proxies care for each other in case of illness, disability, or frailty—is increasingly considered an important pillar for the sustainability of care systems. Especially in a palliative care context, however, the role of a patient’s spouse or relatives is intensified. Family care can be challenging. The concept of burden, then, is well-known. Witnessing caregivers’ challenges may prompt compassionate nurses to undertake actions to reduce a family caregiver’s burden by adjusting tasks or activities. Although timely identification of burden is important, attempts to immediately relieve it may overlook a caregiver’s context and values.

Using a care ethical approach, this theoretical/philosophical paper aims to provide nurses with an alternative perspective on caregiver burden and support. First, to better understand why people persist, we will explain that family care often is not a well-demarcated and chosen activity but an ongoing response ensuing from a relationship. Secondly, we will elaborate on the ambiguities of self-sacrificial family care. Third, we point out that nurses’ support of family caregivers should not primarily be task-oriented but should be tailored to the personal meaning that family care may have for the involved partners or relatives. We also provide some practical suggestions for supporting family caregivers. The paper concludes with a short reflection.

## Background

In palliative care, especially when patients prefer to stay and die at home, the role of their spouse or relatives is pivotal and intensified.^1,2^ Daughter Eva (her fictive story can be read in Table 1) stays at her father’s side until the very end. She provides “family care” which can be defined as the wide range of unpaid care or assistance with activities to someone with a chronic illness, disability, or frailty, given by a person from someone’s direct social network, or one’s partner, sibling, relative, friend, neighbor, or other acquaintance.^5–7^ With aging populations and increase of disabilities, chronic diseases, and frailty pressurizing the sustainability of care, family care is increasingly considered an important pillar of many Western countries’ long-term care systems.^8–10^

**Table 1.** Fictive story of a family caregiver, derived from our research-based graphic novel.

**Figure 1.**
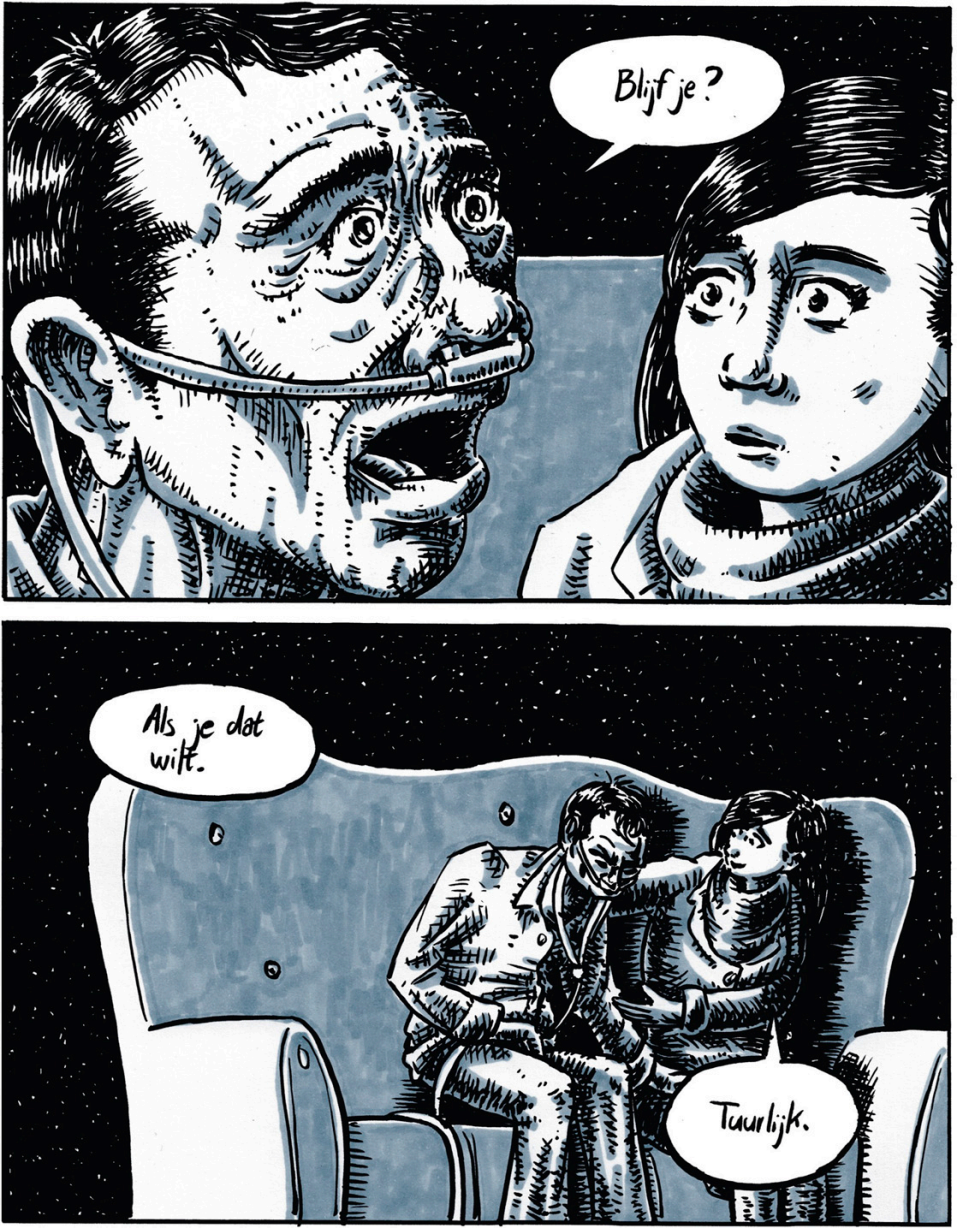
Image of daughter Eva (her story can be read in Table 1), one of the characters in the Dutch graphic novel *Naasten* about family care in a palliative care context. On this page, her father, who suffers from a severe lung condition and is gasping for air, asks her to stay in the middle of the night. “If that’s what you want”, she replies, “Sure.” ©Kranenburg M, Van Ooijen N and Haan MM.* Naasten*. Heverlee: Oogachtend, 2019. (permission was granted for the image)

The Dutch graphic novel *Naasten* shows people’s experiences with family care in palliative care home settings. Fictive, but based on our in-depth interview research.^3,4^ One of the main characters in the novel is daughter Eva (Figure 1). She cares for her father suffering from severe chronic obstructive pulmonary disease (COPD) and tells us about her experiences:
*“My father has been suffering from COPD for a long time*. *Now*, *he is rapidly deteriorating*. *I have a younger sister and an older brother*, *but I’m usually the one who cares for him. I am the oldest daughter*, *you know*, *I feel like I should take care of him. And the others think differently about what’s best for dad*. *Last week*, *he called me in the middle of the night*, *completely panicked and short of breath*. *That was terrifying*. *Of course I rushed off to go to him! You never know if such a phone call is the last one you get from him. And I would not want it any other way - it’s my father. You just do that for each other.*
*It’s tough*, *though*. *We were never that close*, *he was quite distant. He hasn’t been very friendly during his life*, *he has always scared people away. But he raised me*, *to the best of his abilities. And now he needs me*. *So*, *I stop by every day*, *to prepare his food*, *help with medication*, *do some cleaning work. I even help him shower. But having to see him naked… That’s vulnerable*, *you know. I really have to switch a button there – switch it from ‘daughter’ to ‘nurse aid’*, *that helps. I have to keep up.*
*My personal life seems to be in a stand-by mode*. *I have difficulties focusing on my job*. *My boyfriend worries about me*. *And he sometimes complains that we cannot spend much time together. With COPD you never know what will happen next or when the last moment is. So I just do it and keep on going*, *there is no other way.”*

Taking on a caring role for one’s relatives is often expected in current societies and self-evident for many family caregivers.^11^ Our previous study, that included in-depth interviews with partners and adult children of patients receiving palliative care, showed caregivers’ persistent feeling of being called upon to care.^3^ Looking at these caregivers’ stories from an ethical perspective, this being called upon does not seem based on a well-considered choice but presents itself as pre-reflexive. Caregivers often feel a strong urge to act and automatically do so, yet without having been able to thoroughly reflect on why or how. Some people report that they intuitively feel what is needed due to knowing the care receiver so well or having spent so much time together. And then they just *do* it.

At the same time, family care has its challenges. The multidimensional concept of “caregiver burden”^5,12^ is well-known within nursing, given the varied tools to screen for burden in practice.^12,13^ Our previous interview study, from which our story of daughter Eva was derived (Table 1), reported that family caregivers expect themselves to be attentive to the patient first while ignoring their own needs, available all the time, and assertive in managing the caring situation.^3^ In another study, attentiveness, understood as being present and providing emotional support, was regarded to be the most important element in home-based palliative family care but was simultaneously disrupting caregivers’ daily lives.^14^ Caring for a loved one can indeed be overwhelming and all-consuming,^15,16^: it impacts a caregiver’s whole personal realm - limiting living normal daily life^17^ and social engagements,^1,17,18^ - while many caregivers feel unprepared for their caring role.^19^ People, such as daughter Eva, feel limited—“chained” even—in their own lives,^17^ as there is little time for their own families, social lives, hobbies, or work. In short, people tend to regard family care as something self-evident they wish or just have to do, sometimes at considerable personal and social costs.

As nurses spend much time with patients in palliative care settings, they usually are the first and main contact points for patients and their families.^20,21^ Compassion serves as the key motivator for many nurses in their work, stimulating them to turn passion into practice by actively trying to relieve suffering.^22^ It is thus fully understandable that screening is advocated to detect burden at an early stage, especially given the challenges and unmet needs of family caregivers.^12^ Although timely identification of burden is important, attempts to immediately relieve it may overlook a caregiver’s context and values. Assessing caregiver burden needs careful interpretation and consideration of the caregiver’s context.^5,23^ What drives people to provide family care, sometimes at considerable costs? To support sustainable caring relationships between caregivers and patients, we believe it to be important to recognize the complex interplay of underlying motivations for (not) providing family care, especially cultural and societal factors that shape why and how partners or relatives care for each other.^9^ In the remainder of this paper, we will provide another perspective on family caregivers’ experiences and burden, by adopting a care ethical approach.

## Family care as a response to feeling called within a relational and social context

Rather than viewing human beings as independent, utterly self-determining individuals that weigh alternatives and choose freely and deliberately—as the Western ideal of autonomy suggests—^24,25^ care ethicists view people as interdependent and morally motivated by the vulnerability of others.^26,27^ The concept of responsibility, understood as the task to respond to a need, is a corner stone of care ethical thinking: without people taking or accepting this responsibility, there is no care.^25,28^ Due to this mutual interdependence, relationality and contextuality are two additional key elements in care ethics. Care is a *relational* practice which cannot be understood in the abstract but should be considered in the lived experiences of the people that give and receive care within their relationships.^27,29^ These caring relationships are situated in a *context* in which power relations, norms, and expectations play a role.^29^ Our previous interview study also highlighted a normative dimension of family care,^3^ which was incorporated in the stories of our research-based graphic novel (Table 1). In this first section we will explain why family care, from the perspective of care ethics, is not a well-defined task people choose for and can simply withdraw from when it gets too burdensome.

### Family care as a relational practice

Care ethics’ focus on relationality, first, helps us to understand the reality of family care, that is, how most partners or relatives just “find” themselves in the role of family caregiver, due to their specific *relationship* with the one who is ill.^25^ They often accept this responsibility without weighing all possible personal consequences in advance and without actively choosing that role at a particular moment in time.^30^ People seem to just do it. Daughter Eva (Table 1 and Figure 1) gradually stops-by more often, day or night, because her father needs her and “*you just do that for each other.”* Moreover, Barnes explains that it makes little sense to sharply distinguish the group of care receivers and care givers, especially in elderly spousal relationships.^29^ It is hard to tell where care begins or ends, and, as roles often change, to depict who depends on whom. Family care can be framed as “dependency work”: a partner or family member is set free to do other things only if the dependency work of caring is taken over by others.^31^ In that way, both the care receiver and family caregiver are dependent on each other.

In this relational interdependence, overburdening is a serious risk.^12,14^ As indicated in the introduction, family members sometimes persist in caring for their dying loved one at substantial costs, due to interference with their personal wishes, social activities, hobbies, or work.^3,11,14^ Eva (Table 1), for example, experiences increasing difficulties with focusing on her job while also managing her father’s household, care, and medication. Surrounding friends, family, or professionals may react with well-meant advice such as *“don’t forget to think of yourself eh?!”*, as the exhausting caring activities sometimes evoke surprise, awe, or worries by others.^3^ “Thinking of themselves,” however, can be quite difficult for family caregivers. A care ethical perspective helps us to understand why. The crux is in the word “self,” which suggests that there is an autonomous “true self,” independent of the context of the caring relationship, which may be found by disconnecting from one’s relations.^31^ Care-ethicists argue, in contrast, that the self should be understood as ultimately relational.^31^ For family care this means that instead of *having* a terminally ill father, care is about *being* a grown-up child of a terminally ill father.^31^ Family caregivers cannot abandon their relationships and quit being a child or partner altogether. Eva, as her father’s daughter, cannot avoid being called to care.

Of course, people may discard certain caring activities (e.g., bathing one’s parent) or take some time off to catch their breaths, thus in a way temporarily resist a call. Important to acknowledge, however, is that such actions would not erase the overall call to care, due to people’s ongoing alertness to the patient’s needs. Drawing on care ethics, we point-out that family care should not be reduced to a clear-defined task or action with a start and an end, resulting from a well-considered choice.^32^ Rather, it is an ongoing (and often messy) process within a relationship, prompted by attentiveness to each other’s needs.^29^

### The importance of context in understanding how and why people feel called to care

Second, care ethics’ insistence that care is always situated in a specific context is also helpful in understanding family care, especially people’s sometimes immediate and self-nihilating responses to the felt calls. Eva, for example (Table 1), eventually quits her job and tends to neglect her relationship with her boyfriend. Our previous analysis of in-depth interviews with partners and adult children of patients receiving palliative care showed a sense of urgency in these family caregivers’ responses: the décor of deterioration in the palliative care context and the prospect of an approaching death urge people to act now, having only one chance to do so.^3^ Furthermore, one’s capacity to care within one’s family is always affected by specific expectations, existing power relations, and the degree to which caring is valued and supported—not only in the relationship between caregiver and care receiver but also within one’s broader social, cultural, and political context.^29^ Being the oldest daughter, for example, like Eva (Table 1), the darling sibling, or belonging to a family-oriented culture may lead to care expectations.^3^ This implies, again, that living with or caring for a seriously-ill loved one often is not a matter of a straightforward, easy, or even explicit choice.^29^ Rather, actual family care is highly dependent on the specific context in which partners and relatives have to navigate.

By whom and why do people feel called, then? A family caregiver feels connected to (and sometimes torn by) different domains on which they are expected to act. Our aforementioned analysis of interviews with family caregivers showed care may indeed start with being attentive to the patient’s explicit or implicit requests for help, but that well-intending and worried family and friends, one’s own convictions, and healthcare professionals entering normal life may also call upon a caregiver.^3^ These calls, in turn, evoke caregiver responses. To better understand caregivers’ sometimes limitless responses, it is wise to have an eye for the underlying moral dimension of family care. Various studies, some across cultures and different health conditions, have shown the following generic and normative elements—or a combination of them—to be important drivers for people to provide family care, that is, out of love or affection, a sense of obligation or moral contract, reciprocity (e.g., retrospectively “paying back” to one’s parents), but also driven by the pre-existing relationship quality or by one’s family history, values (e.g., solidarity), and relational dynamics.^3,11,14^ Adding to this, we believe it is important for nurses to be aware that such motivations are not purely personal. Whether or not explicitly recognized by caregivers themselves, family care is deeply subject to social norms and expectations (e.g., “seeing one’s parents out,” emphasizing “till death do us part,” or regarding it a women’s responsibility)^9,33^ against the background of wider cultural values and beliefs (e.g., specific norms within one’s family or community, or spiritual and religious beliefs).^9^

## Ambiguity, ambivalence, and self-sacrifice in family care

Family care, thus, involves more than what meets the eye. A complex and normative layer always lies beneath one’s actions or emotions at the surface; and a sense of failure may arise when caregivers’ feelings or experiences conflict with what they believe is expected of a “good caregiver” in their particular social or cultural context.^33^ To better understand family caregivers’ experiences, the concepts of ambiguity and ambivalence are of help. In this second section, we will explain that family care—as a phenomenon—is highly ambiguous, as it realizes certain goods or values while neglecting or suppressing others. That is why family care—as an activity—often leads to ambivalent feelings. We will further explain how burden and self-sacrifice are not necessarily problems that should be solved. In finding an adequate balance for family caregivers, the ambiguities and ambivalences surrounding family care should be openly acknowledged.

### Ambiguity and ambivalence

The phenomenon of family care for someone close has been described as *ambiguous*: some things of value are realized while others are concurrently—inevitably but often unintentionally—oppressed or neglected.^30^ Bereaved caregivers in our previous study were grateful for having persevered and having enjoyed precious “last things” with their loved one, spoke of an intensified and closer relationship, felt honored or proud for having been able to provide family care, but simultaneously reported challenges or exhaustion even.^3^ It is often pointed out that the immense physical, emotional, or psychosocial challenges that family caregivers face while caring for their loved ones require support.^34–37^ Especially within intimate relationships and given its normative complexity, family care can be burdensome. Tragic situations may arise, due to competing demands stemming from one’s different roles:^11^ people may feel torn between being a caregiver while also being a parent, employee, partner, or friend, and experience difficulties (or even exhaustion) in balancing the needs of the patient with one’s own. In responding to a sometimes 24-7 call, they have to deal with expectations from different sources,^3^ as the relational self is connected to several contexts with diverse and sometimes even contrary interests and obligations. Rather than in feeling called per se, the burden of care might lie in the seeming limitlessness to which people feel called, that is, the self-sacrifice that lies in being constantly available and attentive to the patient’s needs first,^3^ and the pressure on certain personal values this causes, like friendship, self-development, or parenthood. Adult children, or the “sandwich generation,” for example, may experience dilemmas in the responsibilities regarding both their parent and their nuclear family.^11^

It may be hard for family caregivers to openly discuss their dilemmas, however. Next to ambiguity, the concept of *ambivalence* has been put forward as helpful in understanding the activity of family care, and particularly the associated caregiver dilemmas and suffering when people’s experiences do not precisely fit within the normative social expectations associated with their role as a family caregiver.^33^ In exploring experiences across death and bereavement, it was shown that the messiness of their loved ones’ dying process meddled with family caregivers’ abilities to meet their initial expectations and desires regarding care. People then may feel regret or shame for not doing or feeling the “right” way, for example, not doing enough, or feeling hateful instead of loving towards the patient.

### Solving the problem of burden?

Whether or not induced by social expectations, family care may lead to self-sacrificial behavior, in which people like Eva (Table 1 and Figure 1) put their personal lives—for example, their job, nuclear family, or social activities—in stand-by mode in favor of what is needed or demanded in the caring situation.^3^ Excessive self-sacrifice would, simply put, be paradoxical as it would lead to a complete loss or even destruction of oneself which, in the end, even leads to not being able to care for one’s loved one anymore.^30^ Supporting a family caregiver then means helping them to find a healthier balance, to prevent them from overstretching or “drowning” themselves and thus being unable to maintain caring, which would lead to less care for patients. Therefore, several tools—some validated and widely used—are advocated to quickly identify perceived caregiver burden,^12,13,23^ one of which related to a classification of caregivers in different risk profiles.^12^

Such screenings and classifications fit well in current healthcare systems, in which evidence-based practice is promoted for early detection of problems and adequate treatment, and nursing skills like critical and analytical thinking, problem solving, and decision-making are deemed vital for efficient care.^38,39^ Concerns, however, have also been raised that this evidence-based paradigm disregards nurses’ tacit and relational knowledge in their patient encounters, as well as the patient’s and nurses’ preferences and values that are important for decision making.^39^ In line with this critique, we suggest that caregivers’ preferences and values are essential to take into account to provide suitable family support. A more in-depth interpretation—which, for example, acknowledges the relational context—is needed when tools or questionnaires are used to identify (potential) burden or risks. Helping family caregivers to find balance is not a simple fix of activities and tasks. A narrowed focus purely on releasing people from their burden would bear the risk of paternalism and would not take into account the ambiguities and ambivalences of family care. Rather, following Gastmans’ thoughts on vulnerability and nursing care,^27^ we believe nurses should provide care that enhances the dignity of both patients *and* their families. Caring for a family caregiver is not always about asking what is to be *done* (or which tasks have to be given up) but rather about how to preserve a person’s dignity as a whole. And thus, caring for family caregivers can be understood as responding to *their* vulnerability. Then, nurses’ support should involve paying attention to all dimensions in which people’s vulnerability affects them—which in the case of older adults would be on a physical, but also a psychological, relational, moral, sociocultural, or existential level.^27,40^

Helping family caregivers, thus, should take into account the trade-off and balancing of different values and expectations—stemming from the various dimensions of their vulnerability—that lie beneath all their caring duties and tasks. Nurses would do well to use their relational powers to enhance such a value clarification, that is, jointly with family caregivers weighing the various values, and acknowledging that contrasting values sometimes lead to dilemmas and tragic situations. Building on Gastmans’ work again, it is this dialogical process that should be shared between nurses and care receivers (in this case family caregivers), to find an appropriate answer to the question (and not necessarily the problem) of burden.^27^ The third section of this paper provides some practical suggestions to engage in such a dialogue with family caregivers.

### Another perspective on self-sacrificial care

Care can be depicted as a gift-sharing process,^14^ especially self-sacrificial care is purely about “giving.”^30^ Van Nistelrooij, however, shows us the multiple meanings of this concept (e.g., giving back, giving in, giving away, and giving up) and explains why self-sacrifice does not have to lead to problematic self-loss.^30^ In her taxonomy of self-sacrifice, sliding from self-limitation to self-destruction, even the extremes are not necessarily problematic but may be viewed as acts that realize a value greater than the self. This is not to imply that family care should be advocated as some heroic act that realizes an ultimate good and thus is always acceptable. Van Nistelrooij warns to always be aware of the boundary between “proper” and “improper” forms of self-sacrifice. But how to pursue balance in family care? Van Nistelrooij defines self-sacrificial care as care provided *“despite the acknowledgment by the self that one will not realize other goods (for instance*, *care for the self)*, *and despite the acknowledgment that the good of this caring is not unambiguous or indisputable”* (p. 286).^30^ So, in ideal circumstances, self-sacrifice would be the result of a conscious balancing act. A certain amount of self-loss can thus be evaluated positively, if family caregivers consciously *acknowledge* that they will not realize other values (e.g., related to their own work, hobbies, family life, or personal goals) and also acknowledge that the intended value or their care is not beyond doubt but can be questioned by themselves or others.

## Implications for nurses’ support

From a care ethical perspective, family care can be viewed as a practice flowing from a relationship within a specific cultural and social context, which has meaning for the people involved. Family care, thus, is no clear-defined task that can or should simply be taken-over in case of burden. Although caring is an integral part of being human, the concrete realization of family care is highly personal, as is finding an adequate balance in fulfilling all motivations and expectations. Next to positive interpretations (giving satisfaction, a sense of purpose or fulfilment, enabling personal growth, etc.),^11^ family care can also have a negative, neutral, or—often—ambiguous or ambivalent meaning. Daughter Eva (Table 1), for example, experiences ambiguities: she finds it simultaneously rewarding and exhausting to give up her values with regard to her job while fulfilling her duty as oldest and darling daughter. She also narrates how they haven’t been very close, as her father always scared people away by not being friendly. Caring may thus be hard on her, possibly leading to ambivalent feelings deemed as “inappropriate” (such as anticipating relief in her father’s death). To prevent family caregivers like Eva from social misrecognition and alienation, we should recognize the complexities of family care in *all* its meanings.^33^

### Supporting family caregivers is about meaning rather than solutions

As we regard consciously finding a balance between contrasting values to be important, we suggest that family caregivers should be enabled to explore and act upon what caring means to them. For nurses and other healthcare professionals, this implies they should regard family caregivers as people within certain relational and social contexts that shape their care and evoke certain behavior. Previous reviews showed the complexity of palliative care nurses’ role,^20,21^ for example, being available, coordinating care, facing clinical challenges in a high workload, while also experiencing the personal impact and helplessness when working with patients in the end stage of serious illness. Dealing with families—for example, with them being demanding or vulnerable—can specifically be a source of stress for nurses.^21^ Grasping the complex interplay of underlying values, family histories and wider social contexts may help nurses in understanding family members and why or how they care.

Furthermore, instant attempts to solve the “problem” of actual burden by changing people’s activities may overlook the inevitable ambiguity in family care, possibly depriving partners and relatives of a potentially self-affirming practice of family care. Moreover, even if burdensome tasks are relieved by practical solutions, family caregivers may still feel called upon: the tragic choices and their consequences still remain. Nurses should therefore not focus on liberating caregivers *from* the situation but support them *in* whatever overwhelms them when providing family care in their specific context.

We strongly suggest that any support for these partners, family members, or other close ones should follow from and be tailored to the personal meaning behind (burdensome) experiences, which could be explored in a dialogue (Table 2). In line with Zarzycki and Morrison’s advice to foster a family caregiver’s critical awareness in a 1:1 conversation between healthcare professional and family caregiver, we would suggest to discuss the values that drive or keep caregivers in their roles.^9^

**Table 2.** Practical suggestions for nurses in supporting family caregivers.

In supporting families, nurses may signal risks of burden. Before immediately taking action, for example, adjusting activities or redistributing tasks to relieve this burden, nurses would do well to first explore what caring (and the possible burden) means for a particular family caregiver. This enables nurses to tailor the type and amount of support to this meaning rather than on tasks or activities. The following practical suggestions may be of help:
(a) Frequently explore the (perhaps various) meanings and accompanying values with caregivers. What does the patient mean to them, and how would they describe their relationship? How do they experience their caring role? What is important in caring for their loved one, and why? What motivates them to care?
(b) Think along with caregivers about how these values might be contrary to one another and what may have to be sacrificed along the way. Which important things or activities are family caregivers unable to do because of their family care? What values are under pressure, and how to weigh these against the gains? Do they experience feelings of failure, and with regard to whom? Give space to feelings of regret or shame regarding their “inappropriate” feelings or actions.
(c) Jointly discuss how (in terms of space, time, activities, etc.) people aim, wish, or feel able to be involved in care. Reflect on (alternative) ways in which their aims (as mentioned under a) can be obtained.
(d) Be aware of self-sacrificial care in which the ambiguities and uncertainties are not acknowledged, for instance, if someone is exhausted but wants to keep on going no matter the costs, or if someone does not see any other option, for example, due to expectations in their family, or practical reasons. Try to understand this in light of the context and relationship of the caregiver and patient. Jointly explore whether the sacrifices are reasonable and healthy. And if not, think about alternatives and how others within or outside their social network can practically help the caregivers in meeting their personal motivations and social expectations.

## Reflection

The suggestions put forward in this paper come with challenges. First, emphasizing the importance of consciously finding a balance between fulfilling all expectations may seem to suggest that it is acceptable when people exhaust themselves as long as this is knowingly done. We want to add that pure exhaustion—even after weighing it in light of other values—may be a signal of excessive and thus improper self-sacrifice.^30^ Then, caregivers are no longer capable of noticing that the “good” of their care is not beyond doubt—all their other roles or activities are put on hold in favor of the goods for the patient, without any space to think about alternatives. Thus, their care would be self-destructive hence disputable. Without doubt, thus, caregiver burnout is a serious risk, prompting authors to advocate support, time-off, and respite care.^9,14^

Furthermore, especially within a palliative care context of intensified care and an approaching death and grief, current caregivers as well as nurses may have neither the energy nor the capacity to thoroughly reflect on motivations or social expectations. We suggest, if possible, to cooperate with other disciplines such as social workers. Putting such deeper layers into words may, however, not be possible or helpful for everyone: due to all ambiguities and ambivalences, people cannot always voice their opinions and preferences, nor are they fully aware of the meaning or consequences of those.^27^ We are aware that our suggestions to help caregivers reflect on meaning and weigh their sacrifices even bears the risk of increasing their burden, as issues become more explicit. Therefore, caution and moderation are required. As is common in advance care planning, we would recommend nurses to invest time in the beginning of a palliative trajectory to explore the family caregiver’s personal situation and ties, together with this caregiver (as suggested in Table 2). This may give nurses an indication of the family caregiver’s social network, familial relationships, and wider social or religious community, and any potential burdening expectations or helpful resources that may spring from them.

Lastly, familial relationships can be very complex and tensed—and some relatives may not be willing to undertake caring activities or may just be absent or hardly involved. Eva’s siblings (Table 1), for example, feel that their dad should be admitted to a caring institution as caring is asking too much of them, whereas Eva wishes to persist in caring for him. From a care ethical perspective, a choice to *not* become a hands-on family caregiver (e.g., to arrange healthcare professionals to help one’s partner shower, or to transfer one’s mother to an institution) can still be understood as “caring” or even “good care” when seen in the particular contexts of the individuals involved and their relationship.^29^ Again, such a decision is not an utterly individual and free choice at a specific moment in time. It is important to understand all choices in the light of the dynamics of the relationship, within the larger context. Whether any decision is “good,” reasonable, or healthy should, in our belief, be discussed jointly with the caregiver. Care ethics offer space for choice. Taking responsibility—crucial for care to even exist—is a moral task after all^25^: people have the freedom to respond to or resist being called upon.

## Summary and conclusion

This paper aimed to provide a care-ethically inspired perspective on family caregiver burden and support in a palliative care context. We explained that family care should not be reduced to clear-defined or well-chosen tasks or activities that can or should simply be taken-over in case of burden. Rather, caring for one’s partner, parent, or other proxy is an ongoing and often messy relational practice, due to being related and therefore called upon, in a particular context. Social or cultural norms and expectations shape how family care both is provided and experienced. We believe caregiver burden may specifically lie in the limitlessness to which people feel called, that is, the self-sacrifice that lies in being constantly available and attentive to the patient’s needs first, and the pressure on certain personal values or other roles this causes (e.g., caring while also being a parent, partner, employee, and friend). Family care, thus, is complex and highly personal, as is finding an adequate balance in fulfilling all of one’s motivations and expectations.

We conclude that nurses would do well to first explore the *meaning* of family care for a specific person, whether positive, negative, or—often—ambiguous or ambivalent, before immediate action is undertaken to relieve burden by adjusting or redistributing tasks to others. If possible, caregiver experiences—and specifically signals of burden or the results from screening tools—should be interpreted in an explorative dialogue with the family caregivers themselves (Table 2). Such a dialogue should keep into perspective the specific caring relationship between the involved people, the person’s social context, and how to balance a person’s values and expectations that shape the impactful experience of caring for a dying loved one.

We are fully aware, however, that this paper describes an ideal, whereas the actual world of family caregivers and of nurses’ support may be tough and messy, especially in acute, brief, or intensive caring processes. Family caregivers may neither have time nor the capacity to thoroughly reflect on what caring means for them. Notwithstanding, we suggest to carefully tailor support to what lies beneath burdensome experiences. Nurses, compassionate as they often are, do not have to strive to liberate family caregivers *from* the situation but should support them *in* whatever overwhelms or drives them in standing-by their loved ones until the end.

## References

[1] Morris SM King C Turner M , et al. Family carers providing support to a person dying in the home setting: a narrative literature review. Palliat Med 2015; 29: 487-495. 25634635 10.1177/0269216314565706 PMC4436280

[2] Vermorgen M Vandenbogaerde I Van Audenhove C , et al. Are family carers part of the care team providing end-of-life care? A qualitative interview study on the collaboration between family and professional carers. Palliat Med 2020. 10.1177/0269216320954342 32928056

[3] Haan MM Olthuis G van Gurp JL . Feeling called to care: a qualitative interview study on normativity in family caregivers’ experiences in Dutch home settings in a palliative care context. BMC Palliat Care 2021; 20: 183–215. 34837984 10.1186/s12904-021-00868-2 PMC8626934

[4] Haan M Olthuis G Boenink M , et al. Bridging comic art and research: lessons from an interdisciplinary collaboration project in a palliative care context. Med Humanit 2024. 10.1136/medhum-2023-012750 38453454

[5] Choi S Seo J . Analysis of caregiver burden in palliative care: an integrated review. Nurs Forum 2019; 54: 280–290. 30737798 10.1111/nuf.12328

[6] Alam S Hannon B Zimmermann C . Palliative care for family caregivers. J Clin Oncol 2020; 38: 926–936. 32023152 10.1200/JCO.19.00018

[7] Plothner M Schmidt K de Jong L , et al. Needs and preferences of informal caregivers regarding outpatient care for the elderly: a systematic literature review. BMC Geriatr 2019; 19: 82. 30866827 10.1186/s12877-019-1068-4 PMC6417014

[8] Elayan S Bei E Ferraris G , et al. Cohort profile: the ENTWINE iCohort study, a multinational longitudinal web-based study of informal care. PLoS One 2024; 19: e0294106. 38236932 10.1371/journal.pone.0294106 PMC10796045

[9] Zarzycki M Morrison V Bei E , et al. Cultural and societal motivations for being informal caregivers: a qualitative systematic review and meta-synthesis. Health Psychol Rev 2023; 17: 247–276. 35081864 10.1080/17437199.2022.2032259

[10] Verbakel E . How to understand informal caregiving patterns in Europe? The role of formal long-term care provisions and family care norms. Scand J Publ Health 2018; 46: 436–447. 10.1177/1403494817726197 PMC5989248 28823224

[11] Zarzycki M Seddon D Bei E , et al. Why do they care? A qualitative systematic review and meta-synthesis of personal and relational motivations for providing informal care. Health Psychol Rev 2023; 17: 344–376. 35383541 10.1080/17437199.2022.2058581

[12] Ullrich A Bergelt C Marx G , et al. The CAREPAL-8: a short screening tool for multidimensional family caregiver burden in palliative care. BMC Palliat Care 2024; 23: 195. 39095830 10.1186/s12904-024-01480-w PMC11295689

[13] Pop RS Payne S Tint D , et al. Instruments to assess the burden of care for family caregivers of adult palliative care patients. Int J Palliat Nurs 2022; 28: 80–99. 35446673 10.12968/ijpn.2022.28.2.80

[14] Sarradon-Eck A Mathiot A Holmes SM , et al. The moral dimensions of family caregiving for patients with advanced cancer: a qualitative study. Eur J Cancer Care 2023; 2023: 1–9.

[15] Breen LJ Aoun SM O’Connor M , et al. Family caregivers’ preparations for death: a qualitative analysis. J Pain Symptom Manag 2018; 55: 1473-1479. 10.1016/j.jpainsymman.2018.02.018 29499235

[16] Robinson CA Bottorff JL McFee E , et al. Caring at home until death: enabled determination. Support Care Cancer 2017; 25: 1229-1236. 27924357 10.1007/s00520-016-3515-5

[17] Martín JM Olano-Lizarraga M Saracíbar-Razquin M . The experience of family caregivers caring for a terminal patient at home: a research review. Int J Nurs Stud 2016; 64: 1-12. 27657662 10.1016/j.ijnurstu.2016.09.010

[18] Van Roij J Brom L Youssef-El Soud M , et al. Social consequences of advanced cancer in patients and their informal caregivers: a qualitative study. Support Care Cancer 2019; 27: 1187-1195. 30209602 10.1007/s00520-018-4437-1 PMC6394690

[19] Funk L Stajduhar K Toye C , et al. Part 2: home-based family caregiving at the end of life: a comprehensive review of published qualitative research (1998-2008). Palliat Med 2010; 24: 594-607. 20576673 10.1177/0269216310371411

[20] Sekse RJT Hunskår I Ellingsen S . The nurse’s role in palliative care: a qualitative meta‐synthesis. J Clin Nurs 2018; 27: e21–e38. 28695651 10.1111/jocn.13912

[21] Clayton M Marczak M . Palliative care nurses’ experiences of stress, anxiety, and burnout: a thematic synthesis. Palliat Support Care 2023; 21: 498–514. 35706143 10.1017/S147895152200058X

[22] van der Cingel M Brouwer J . What makes a nurse today? A debate on the nursing professional identity and its need for change. Nurs Philos 2021; 22: e12343. 33450124 10.1111/nup.12343

[23] Onega LL . The modified caregiver strain index (MCSI). J Gerontol Nurs 2013; 33: 19–26.

[24] Verkerk MA . The care perspective and autonomy. Med Health Care Philos 2001; 4: 289–294. 11760228 10.1023/a:1012048907443

[25] van Nistelrooij I Visse M . Me? The invisible call of responsibility and its promise for care ethics: a phenomenological view. Med Health Care Philos 2019; 22: 275–285. 30327903 10.1007/s11019-018-9873-7 PMC6499747

[26] Nortvedt P Hem MH Skirbekk H . The ethics of care: role obligations and moderate partiality in health care. Nurs Ethics 2011; 18: 192–200. 21372232 10.1177/0969733010388926

[27] Gastmans C . Dignity-enhancing nursing care: a foundational ethical framework. Nurs Ethics 2013; 20: 142–149. 23466947 10.1177/0969733012473772

[28] Tronto JC . Moral boundaries. A political argument for an ethic of care. New York: Routledge, 1993.

[29] Barnes M . Care in everyday life: an ethic of care in practice. Policy Press, 2012.

[30] van Nistelrooij I . Self-sacrifice and care ethics. Sacrifice in modernity: community, ritual, identity. Brill, 2020, pp. 270–287.

[31] van Nistelrooij I Visse M Spekkink A , et al. How shared is shared decision-making? A care-ethical view on the role of partner and family. J Med Ethics 2017; 43: 637–644. 28356489 10.1136/medethics-2016-103791

[32] Tronto JC . Creating caring institutions: politics, plurality, and purpose. Ethics Soc Welfare 2010; 4: 158–171.

[33] Broom A Parker RB Kenny K . Authenticity, ambivalence and recognition in caring at the end of life and beyond. Soc Sci Med 2019; 239: 112554. 31542650 10.1016/j.socscimed.2019.112554

[34] Ahn S Romo RD Campbell CL . A systematic review of interventions for family caregivers who care for patients with advanced cancer at home. Patient Educ Counsel 2020; 103: 1518–1530. 10.1016/j.pec.2020.03.012 PMC7311285 32201172

[35] Totman J Pistrang N Smith S , et al. ‘You only have one chance to get it right’: a qualitative study of relatives’ experiences of caring at home for a family member with terminal cancer. Palliat Med 2015; 29: 496-507. 25634637 10.1177/0269216314566840

[36] Oechsle K . Current advances in palliative & hospice care: problems and needs of relatives and family caregivers during palliative and hospice care—an overview of current literature. Med Sci 2019; 7: 43. 10.3390/medsci7030043 PMC6473856 30871105

[37] Tarberg AS Kvangarsnes M Hole T , et al. Silent voices: family caregivers’ narratives of involvement in palliative care. Nurs Open 2019; 6: 1446-1454. 31660172 10.1002/nop2.344 PMC6805263

[38] Rasmussen P Henderson A McCallum J , et al. Professional identity in nursing: a mixed method research study. Nurse Educ Pract 2021; 52: 103039. 33823376 10.1016/j.nepr.2021.103039

[39] Kuijper S Felder M Clegg S , et al. “We don’t experiment with our patients!” An ethnographic account of the epistemic politics of (re) designing nursing work. Soc Sci Med 2024; 340: 116482. 38064819 10.1016/j.socscimed.2023.116482

[40] Gastmans C Sala R Sanchini V . The concept of vulnerability in aged care: a systematic review of argument-based ethics literature. 2022. 10.1186/s12910-022-00819-3 PMC9379886 35974362

